# A Case of *Rahnella aquatilis* Bacteraemia After the Receipt of an Unlicensed Intravenous Vitamin Infusion, the First to be Reported in the UK

**DOI:** 10.1155/crdi/8351893

**Published:** 2025-11-14

**Authors:** Amelia Benjamin, Partho Roy, Natalie Pedersen, Madhuri Vidwans

**Affiliations:** ^1^West Hertfordshire Teaching Hospitals NHS Trust, Hertfordshire, UK; ^2^UK Health Security Agency (UKHSA), North London Health Protection Team (HPT), London, UK

## Abstract

*Rahnella aquatilis* is a Gram-negative bacterium commonly found in the environment. Previously associated with food spoilage, it has in the past been responsible for causing infection in humans, usually as an opportunistic pathogen. We report a case of a previously healthy female who was diagnosed with *R. aquatilis* bacteraemia. Careful history-taking confirmed she had received an intravenous infusion of presumed-contaminated vitamins immediately prior to presentation. As the global beauty-industry continues to expand, we should be vigilant of future similar cases.

## 1. Introduction

*Rahnella aquatilis* is a facultatively anaerobic Gram-negative bacillus that is a member of the order *Enterobacterales* and member of the family Yersininaceae [1]. Typically isolated from the environment (often fresh water hence ‘*aquatilis*') [1, 2], it has also been described as a causative pathogen in bloodstream [3–6] and urinary tract infections [2, 7] usually, but not always, in the setting of an immunocompromised host [8]. Endocarditis has also been reported in a paediatric patient with congenital heart disease [9]. The full clinical spectrum of disease caused by this organism is not yet understood, due to the paucity of literature describing such cases. However, it seems that the pathogen could cause overwhelming sepsis [3, 6, 10].

We describe the case of an immunocompetent female who was diagnosed with a *R. aquatilis* bacteraemia after presenting with features of septic shock. A thorough history revealed that the patient was likely to have acquired the bacteraemia from a vitamin infusion administered by a freelance beauty practitioner working in a beauty clinic. The case, the first to be reported in the UK, highlights the risks of bloodstream infections associated with such procedures.

## 2. Case Report

A 39-year-old immunocompetent female, with no past medical history except for breast augmentation surgery 16 years previously, presented to the emergency department (ED) with an acute history of right-sided abdominal pain, vomiting and diarrhoea. She also described dysuria, but no chest pain or significant shortness of breath. On examination, she exhibited signs of sepsis, with low blood pressure (91/41 mmHg), tachycardia (heart rate 128 beats per minute) and fever (temperature 40.7°C). Oxygenation and respiratory rate were within normal limits.

Bloods on admission are shown in Table 1. Of note, the patient was leucopenic on admission, primarily showing neutropenia and lymphopenia. It is unclear whether these results were spurious; the neutropenia resolved on bloods sent the following day (although this may have been reactive to the bloodstream infection). Computed tomography (CT) scan pulmonary angiogram abdomen and pelvis revealed bilateral lower lobe pulmonary segmental consolidation only. She was commenced on empirical antibiotics with piperacillin-tazobactam 4.5 g 8 hourly and a single dose of gentamicin. Her clinical status quickly deteriorated, and she required inotropic support in an intensive care setting. At this point, the medical team responsible for her care made the provisional diagnosis of urosepsis.

The patient had two sets of peripheral blood cultures (aerobic and anaerobic bottle) taken within the first 4 h of her admission. After initial processing, both demonstrated the presence of Gram-negative bacilli on gram staining (the first set in both anaerobic and aerobic bottles, with growth at 11 h, and the second set in the aerobic bottle only, with growth at 22 h). On the same evening of the initial gram stain result, using matrix-assisted laser desorption/ionisation (MALDI-TOF), the organism grown in both sets of blood culture bottles was identified as *R. aquatilis* (MALDI score of 2.32 and 2.29 for the organism in the first and second blood culture, respectively). The MALDI-TOF software package used was the Bruker Maldi-HDT IVD Sirius.

At the point of the bacterial species identification, a review of the available literature demonstrated that *R. aquatilis* possesses a chromosomally encoded Ambler Class A extended spectrum beta lactamase (ESBL) enzyme (RAHN-1) [11]. Given this information, the critical condition of the patient, and the confirmed growth of *R. aquatilis* in blood cultures, piperacillin-tazobactam was empirically escalated to meropenem 1 g 8 hourly (due to concern that the former antibiotic is not reliable in treating other ESBL-producing *Enterobacterales* bloodstream infections) [12], pending the antibiotic sensitivity profile of the organism to become available.

Antimicrobial sensitivity was performed manually using the Kirby–Bauer method. Antimicrobial susceptibility testing (AST) profiles were determined using disc diffusion on Mueller–Hinton agar (Oxoid). The in vitro sensitivity profile is shown in Table 1. Zone disc diameters were interpreted using the European Committee on Antimicrobial Susceptibility Testing (EUCAST)'s methodology. Our laboratory's first line *Enterobacterales* AST panel was performed on the first blood culture growth of *R. aquatilis*; the organism grown from the second set was tested against the ‘urinary *Enterobacterales*' panel. The urinary panel was used owing to the rarity of the organism and the team's suspicion of the bacteraemia being urinary in origin. It should be noted that whilst most of some antibiotics in the urinary panel are inappropriate for bacteraemia (i.e., pivmecillinam and nitrofurantoin), they may have use in other cases, if needing to treat only urinary tract infection. The isolate was sensitive to amoxicillin-clavulanic acid when tested against the first line antibiotic panel but resistant to the same antibiotic in the second panel. This is likely due to differences in zone diameter interpretation for each panel.

The isolate was confirmed as being an extended-spectrum beta lactamase (ESBL) producer, using the MAST D72C AmpC ESBL CPE detection set.

*Rahnella aquatilis* is ubiquitous in the environment, and whilst it remains an unusual pathogen in immunocompetent adults, it has previously been associated with line infections in these patients [13]. Considering this, a more detailed exposure history was taken. It transpired that on the afternoon of her presentation to hospital; the patient had visited a beauty clinic where she had received an intravenous infusion of vitamins administered by a freelance practitioner. The patient reported that she had felt well prior to visiting the clinic; however, during the infusion of the second vial of vitamins, she had started to vomit and developed wheeze and shortness of breath. Following this, she returned home, where she continued to vomit and have diarrhoea, before presenting to the ED. The patient denied recent travel or the consumption of unusual or uncooked foods within the preceding 48 h of her admission; however, a detailed account of every food/drink ingested over the days prior to her admission was not taken.

Meropenem was continued; the patient clinically improved, and 7 days after admission to hospital her care was stepped down to a medical ward. The patient did not have an echocardiogram owing to her marked response to antibiotic therapy. Antibiotics were switched to oral ciprofloxacin 500 mg 12 hourly, and she completed a 10-day course of antibiotics in total.

The case was referred to the United Kingdom Health Security Agency (UKHSA), who together with the Care Quality Commission (CQC) and the local authority Health and Safety Team investigated both the practitioner and the beauty clinic. During this investigation, the practitioner was referred to both the General Dental Council (for inappropriate use of protected titles) and the Advertising Standards Agency (for the cosmetic procedures advertised). UKHSA was unfortunately unable to trace the batch of vitamin vials for further testing— thus their contamination remains a presumption—nonetheless, we believe it to be the most likely source of the patient's bacteraemia.

## 3. Discussion and Review of the Literature Describing Cases of Human Infection

Throughout their lifetime, humans are likely to have frequent exposure to *Rahnella* species; high concentrations have been discovered in minced meat, freshwater fish and dairy produce [10]. The bacterium is a potential public health concern; there are reports of it producing the gene for *Escherichia coli* heat labile toxin, and it is associated with dangerous levels of histamine in fish products. The bacterium has been implicated in the spoilage of food [14]; there are also hypothesised (but microbiologically unconfirmed) accounts of it contaminating bags of total parenteral nutrition (TPN) [15].

We conducted a brief review of the literature describing invasive infection caused by *R. aquatilis* in humans. Two separate searches were conducted, the first used the terms ‘*Rahnella aquatilis*' and ‘invasive infection,' and the second ‘*Rahnella aquatilis*' and ‘human infection.' Both searches returned a combined total of 21 papers. Invasive infection was defined as the presence of bacteria in normally sterile sites (thus would include bacteraemia, invasive skin/soft tissue infection, pyelonephritis and meningitis and exclude nonbacteraemic pneumonia, urinary tract infection or superficial wound infection). Studies that did not describe invasive *R. aquatilis* infection in humans (*n* = 5) or not written in English were excluded (*n* = 1). Fifteen papers met these criteria and are described in Table 2.

Eight of the 16 patients with invasive *R. aquatilis* infection were immunocompromised. The source of infection in seven patients was related to a possible intravenous line or port infection; three patients' infections were linked to contamination of infusions. Three patients had an entirely unclear source of their infection. Therapy varied in choice of antimicrobial and duration; all patients survived, however.

There are confirmed reports of *R. aquatilis* causing sepsis in immunocompetent adults after contaminating vitamin infusion vials that have subsequently been infused intravenously [3]. It may be assumed that our exposure to the bacterium will continue to increase as the number of people seeking vitamin infusions increases.

*Rahnella* species has been discovered in plant roots and rhizospheres [22]. It possesses several virulence factors, including endotoxins [23] and signalling molecules to facilitate quorum sensing, surface motility and biofilm formation [24], amongst others [25].

Previous work has demonstrated that *R. aquatilis* possesses a chromosomally encoded extended-spectrum Ambler class A beta-lactamase (RAHN-1) [11]; therefore, penicillins and cephalosporins might not be effective for treatment and should be avoided if possible (although cephalosporins have been successfully used as therapy in some cases) [13]. Carbapenems, co-trimoxazole and quinolones can be appropriate empirical therapy. *Rahnella* species grow well under standard conditions [2]; previously (prior to being listed within commercial API systems/identified through the MALDI-TOF) [3], they have been misidentified as *Enterobacter agglomerans*.

According to data extracted from UKHSA's Second Generation Surveillance System, from the start of 2021 to September 2023 (when UKHSA's investigation into this case began), *Rahnella* species or *R. aquatilis* had been isolated from clinical samples on 29 occasions in NHS hospitals in England and Wales, including blood, sputum, cerebrospinal fluid (CSF) and muscle biopsy. There is no suggestion that this is in exceedance of numbers of cases expected, or that there are links between these infections. These cases are likely to be due to the increased use of MALDI-TOF in NHS laboratories, rather than a sudden increase in infections caused by *Rahnella* species.

One limitation of this case is its inability to prove that the source of this patient's bacteraemia was the infused vitamin vials, as these were unable to be traced and subsequently sampled. Nonetheless, the timing of her clinical deterioration would fit with the infusion being contaminated with *R. aquatilis*, and similar cases have been reported in the literature. In view of contaminated foods being a theoretical source of *R. aquatilis*, in retrospect, an itemised account of her recent food/drink consumption may have been useful, to exclude this as a potential source of her bacteraemia.

This case report highlights the need to consider *Rahnella* species as significant opportunistic pathogens; they can cause fulminant sepsis in immunocompetent patients. As the United Kingdom's beauty industry develops, with increasing numbers of (both licenced and unlicenced) intravenous vitamin infusions available for use, *Rahnella aquatilis* and other environmental bacteria are likely to become more well-recognized as potential human pathogens. Furthermore, the synergistic work of clinical microbiologists and public health practitioners is highlighted by this case report.

## Figures and Tables

**Table 1:** Blood test and microbiology results from the patient's first 2 days post admission to hospital.

		Day 1 admission	Day 2 admission
Bloods	Haemoglobin (g/dL)	136	119
	White blood cell count (× 10^9^/L)	1.5	6.90
	Neutrophil count (× 10^9^/L)	1.32	6.53
	Lymphocyte count (× 10^9^/L)	0.18	0.30
	Platelets (× 10^9^/L)	271	215
	Sodium (mmol/L)	140	139
	Potassium (mmol/L)	3.6	3.8
	Creatinine (mmol/L)	133	130
	eGFR (mL/min)	43	45
	Magnesium (mg/dL)	0.58	0.51
	Bilirubin (μmol/L)	15	20
	Alanine transferase (ALT) (U/L)	94	174
	ALP (IU/L)	72	54
	GGT (U/L)	230	244
	CRP (mg/L)	< 5	28
	Adjusted calcium (mmol/L)	2.21	1.99

Peripheral blood cultures		Blood culture set sent on admission	Blood culture set sent 4 h later
		Both aerobic and anaerobic bottles grew Gram-negative bacilli identified as *Rahnella aquatilis* on MALDI-TOF.	Aerobic bottle grew Gram-negative bacilli identified as *Rahnella aquatilis* on MALDI-TOF.
		Sensitivity profile:	Sensitivity profile:
		Gentamicin S	Gentamicin S
		Amoxicillin R	Amoxicillin R
		Amoxicillin-clavulanic acid S	Amoxicillin-clavulanic acid R
		Ciprofloxacin S	Ciprofloxacin S
		Cefuroxime R	Cefixime I
		Cefotaxime S	Cefalexin I
		Cefpodoxime R	Cefpodoxime R
		Meropenem S	Piperacillin/tazobactam S
		Piperacillin/tazobactam S	Trimethoprim I
		Ceftazidime S	Norfloxacin I
		Ertapenem S	Pivmecillinam I
		Temocillin I	Nitrofurantoin I

Urine MCS		0 white cells, negative nitrites, nil bacterial growth.	

Abbreviations: I = Sensitive at increased doses and R = Resistant, S = Susceptible.

**Table 2:** Literature review describing case reports describing other cases of invasive *R. aquatilis* infection.

Authors, year of publication	Patient age and sex	Immunocompetency	Site of isolated organism	Site of infection	Presumed or confirmed source of infection	Antibiotic treatment	Patient outcome
Goubau et al. 1988 [6]	42F	Immunocompromised (Dx ALL, receiving chemotherapy, T2DM)	Blood culture	Blood culture	Unclear. Patient did have a line in situ.	Vancomycin (500 mg four-times-daily) and ceftazidime (2 g three times daily). U/K duration, U/K if line removed.	Survived

Hoppe et al. 1993 [4]	7M	Immunocompromised (Dx neuroblastoma, receiving chemotherapy, 131 I-MIBG, +conditioning regimen for autologous stem cell transplantation.	Blood culture.	Blood culture.	Hickmann line presumed source of infection.	Amikacin and imipenem-cilastin. U/K dose, U/K duration, line not removed.	Survived.

Funke and Rosner 1995 [16]	21M	HIV-infection, CD4 count 40/μL, intravenous drug use.	Blood culture.	Blood culture.	Unclear. Possible use IV drugs through IV cannula when outside the hospital, that were contaminated from a water source.	Peripheral intravenous cannula replaced. Treated with 5 days intravenous ciprofloxacin 400 mg twice-daily, followed by 9 days oral therapy with 500 mg three times daily.	Survived.

Oh and Tay 1995 [5]	Case 1: 48M Case 2: 57M	Case 1: Diabetes mellitus. Unclear control. Case 2: Recent diagnosis of laryngeal carcinoma. No chemotherapy received prior to bacteraemia diagnosis.	Blood culture.	Blood culture.	Case 1: Unclear. Concurrent ileocaecal abscess. *R*. *aquatilis* not cultured from pus from the abscess. Case 2: Unclear. Emergency tracheostomy performed 2 days prior to bacteraemia diagnosis.	Case 1: Intravenous amoxicillin-clavulanic acid 1.2 g 8 hourly (14 days), with gentamicin 60 mg 8 hrly (19 days). Case 2: 7 days ceftriaxone 1 g 24 hrly, followed by 5 day course gentamicin 60 mg 8 hrly.	Case 1: Survived Case 2: Survived.

Matsukura et al. 1996 [9]	11 month F	Immunocompetent, CHD.	Blood culture.	Infective endocarditis.	Unclear, possibly IV cannula.	Netilmicin (U/K dose and duration) + ceftazidime 200 mg/kg/day (11 days), followed by 31 days of 100 mg/kg per day (6 weeks total). IV cannula removal.	Survived.

Caroff et al. 1998 [15]	32F	Immunocompetent	Blood culture.	Bacteraemia.	Presumed source contaminated TPN vials. Not confirmed with sampling of TPN vials.	No antibiotic treatment.	Survived.
	61M	Renal cell carcinoma with recent radical nephrectomy (no chemo- or radiotherapy reported).	Blood culture.	Bacteraemia.		NR.	Survived.

Chang et al. 1999 [3]	26M	Immunocompetent.	Blood culture.	Bacteraemia.	Contaminated intravenous vitamin infusion (microbiologically confirmed).	Three days ceftriaxone and imipenem, (U/K dose).	Survived.

Carinder et al. 2005 [17]	46M	ALL, receiving chemotherapy.	Blood culture.	Bacteraemia.	Unclear, possible Hickmann line.	4 weeks TZP + gentamicin (U/K dose). U/K if line removed.	Survived.

Tash 2005 [2]	76M	Immunocompetent.	Blood culture.	Bacteraemia.	Urine.	7 days levofloxacin.	Survived.

Gaitán and Bronze 2010 [8]	27F	Sickle cell anaemia.	Blood culture.	Bacteraemia.	Peripheral intravenous cannula.	14 days ciprofloxacin.	Survived.

Kuzdan et al. 2015 [18]	30 day M	Prematurity (27 weeks gestation).	Blood culture.	Bacteraemia.	Unclear.	21 days meropenem and amikacin (U/K dose).	Survived.

Lee et al. 2019 [19]	58F	IDC of breast, receiving chemotherapy.	Blood culture.	Bacteraemia.	Chemoport.	Treated with combination of cefotiam, cefixime, piperacillin-tazobactam and ciprofloxacin (U/K doses) for 21 days, followed by chemoport removal.	Survived.

Roeder et al. 2021 [13]	37M	Immunocompetent	Blood culture.	Bacteraemia.	Unclear- possibly TPN, possibly PICC line.	10 days cefepime 2 g (U/K dose frequency), followed by ceftriaxone 2 g once-daily. PICC line removed.	Survived, full recovery.

Williams et al. 2025 [20]	74M	Type 2 diabetes mellitus.	Vitreous fluid.	Endophthalmitis.	Unclear.	Core vitrectomy, topical ciprofloxacin and intravitreal vancomycin and ceftazidime (U/K dose).	Survived.

Nicod et al. 2025 [21]	33M	Immunocompetent.	Purulent joint fluid from PIPJ, carpal tunnel, flexor tendon sheaths.	Arthritis (poly-microbial).	Cat bite.	Amoxicillin–clavulanic acid 875 mg three times daily for 6 weeks.	Survived, full recovery hand function.

Abbreviations: 131 I-MIBG = radioactive iodine MIBG, AL = acute lymphoblastic leukaemia, CHD = congenital heart disease, Dx = diagnosis, F = female, G = grams, HIV = human immunodeficiency virus, IDC = invasive ductal carcinoma, IV = intravenous, M = male, mg = milligrams, NR = not reported, PIPJ = proximal interphalangeal joints, TPN = total parenteral nutrition, and U/K = unknown.
